# The different course of alcoholic and idiopathic chronic pancreatitis: A long-term study of 2,037 patients

**DOI:** 10.1371/journal.pone.0198365

**Published:** 2018-06-08

**Authors:** Lu Hao, Li-Sheng Wang, Yu Liu, Teng Wang, Hong-Lei Guo, Jun Pan, Dan Wang, Ya-Wei Bi, Jun-Tao Ji, Lei Xin, Ting-Ting Du, Jin-Huan Lin, Di Zhang, Xiang-Peng Zeng, Wen-Bin Zou, Hui Chen, Ting Xie, Bai-Rong Li, Zhuan Liao, Zhi-Jie Cong, Zheng-Lei Xu, Zhao-Shen Li, Liang-Hao Hu

**Affiliations:** 1 Department of Gastroenterology, Changhai Hospital, The Second Military Medical University, Shanghai, China; 2 Digestive Endoscopy Center, Changhai Hospital, The Second Military Medical University, Shanghai, China; 3 Department of Gastroenterology, The Second Clinical Medical College (Shenzhen People’s Hospital), Jinan University, Guangdong, China; 4 Department of Gastroenterology, Zhongda Hospital, Southeast University, Nanjing, China; 5 Department of Gastroenterology, Air Force General Hospital, Beijing, China; 6 Department of General Surgery, Renji Hospital, Shanghai Jiaotong University, Shanghai, China; Centro Nacional de Investigaciones Oncologicas, SPAIN

## Abstract

**Background:**

Chronic pancreatitis (CP) is a chronic inflammatory disease of the pancreas. This study aimed to compare the natural course of alcoholic chronic pancreatitis (ACP) and idiopathic chronic pancreatitis (ICP).

**Methods:**

CP patients admitted to our center from January 2000 to December 2013 were enrolled. Characteristics were compared between ACP and ICP patients. Cumulative rates of diabetes mellitus (DM), steatorrhea, pancreatic stone, pancreatic pseudocyst, biliary stricture, and pancreatic cancer after the onset and the diagnosis of CP were calculated, respectively. The cumulative rates of DM and steatorrhea after diagnosis of pancreatic stone were also calculated.

**Results:**

A total of 2,037 patients were enrolled. Among them, 19.8% (404/2,037) were ACP and 80.2% (1,633/2,037) were ICP patients. ACP and ICP differs in many aspects, especially in gender, age, smoking, complications, morphology of pancreatic duct, and type of pain. The development of DM, steatorrhea, PPC, pancreatic stone, and biliary stricture were significantly earlier and more common in ACP patients. No significant difference was observed for pancreatic cancer development. There was a rather close correlation between exocrine/endocrine insufficiency and pancreatic stone in ACP patients, which was much less correlated in ICP patients.

**Conclusion:**

The long-term profile of ACP and ICP differs in some important aspects. ACP patients usually have a more severe course of CP. These differences should be recognized in the diagnosis and treatment of CP.

## Introduction

Chronic pancreatitis (CP) is a chronic inflammatory disease of the pancreas, which leads to a progressive and irreversible destruction of pancreatic parenchyme and ductal structures[1, 2]. Alcoholic chronic pancreatitis (ACP) patients were those abused alcohol; while idiopathic chronic pancreatitis (ICP) patients were those had no recognized causes of CP.

Since 1963 several classifications of CP have been introduced [3–7]. These classifications were mainly concerned with the distinction between acute pancreatitis and CP. Moreover, they focused primarily on ACP and only marginally considered the nonalcoholic types. Ammann et al. were among the first to analyze the longitudinal course of CP based on 287 CP patients in 1980’s. They correlated it with cause, and found that excessive alcohol use was associated with a more severe course [8,9]. ACP is characterized by recurrent episodes of acute pancreatitis in early stages and by progressive pancreatic dysfunction. In its later stages, pain relief occurs spontaneously in some of them [8]. Moreover, they first described nonalcoholic, idiopathicCP characterized first by onset of symptoms late in life, and second by a different clinical course compared with ACP [9,10].

The above studies focused on the natural course of ACP and ICP patients helped us better understand CP. However, these studies were carried out decades ago, with relative small sample size. With the change of lifestyle habits in recent years, the proportion of ACP and ICP have changed [11–14], and the differences between course of ACP and ICP are still unclear. There’s still lack of studies based on large sample size of CP patients and longtime of follow-up, especially in recent years. Thus, study should be performed to explore the difference of natural course of ACP and ICP in a larger scale and longer follow-up time.

The study aimed to evaluate epidemiological features, initial manifestations, natural course and complications, and compare them between alcoholic and idiopathic CP. This may help us to choose the suitable treatment strategy according to the etiologies of CP.

## Materials and methods

### Patients and database

Since the 1990s, an electronic medical record system (GOODWILL Inc., Beijing, China) has been used in Changhai Hospital, which has facilitated several studies on CP [2,12,15–20]. In order to track changes consistently throughout the course of CP and facilitate the evaluation and study of CP, a dedicated database, the CP Database (version number 2.1, YINMA Information Technology Inc., Shanghai, China) was established in 2005 to collect clinical data of CP patients who were admitted to our center. Data from January 2000 to December 2004 were retrospectively collected according to the electronic medical record system and complemented through telephone, letter, and e-mail inquiries. Data were prospectively collected since January 2005. The following information was documented in detail: demographic data (age, sex, birthplace, et al), course of CP, medical history, history of other diseases, tobacco and alcohol consumption, family history of pancreatic diseases and diabetes mellitus (DM), laboratory and imaging findings, and treatment strategy.

The database system was set to remind the investigators to call patients for clinical checkups. Aside from visits due to complaints of discomfort related to CP, all patients were periodically (annually at least) recalled for clinical checkup and investigations. Ultrasound, magnetic resonance imaging (MRI), or computed tomography (CT) was selected as an evaluation modality during each follow-up visit. An evaluation of each revisit or evaluation via telephone inquiries for patients who did not return to our center was added to the CP database. In December 2013, we contacted all the patients in our database for a final evaluation, except those who were lost to follow-up or died. The duration of follow-up is defined as the duration from the onset of CP to the date of the last personal contact, death, or end of follow-up (December 2013), whichever came first.

**Fig 1:**
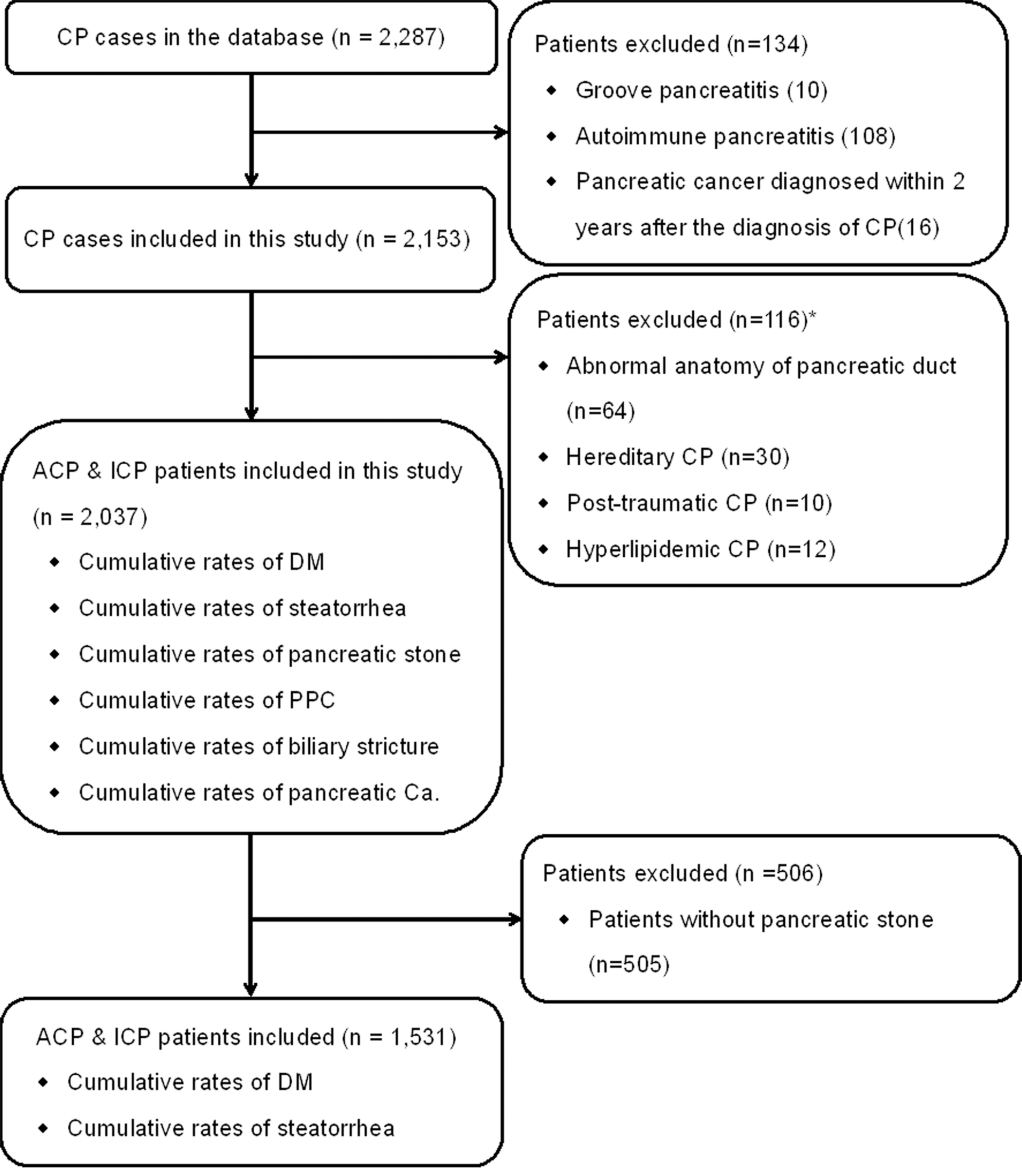
Flow diagram of patients enrollment and study design. *Abnormal anatomy of pancreatic duct includes pancreas divisum and anomalous pancreatico-biliary junction [23]. Hereditary CP refers to 2 first-degree relatives or ≥3 second-degree relatives, in ≥2 generations with recurrent acute pancreatitis and/or CP, for which there were no precipitating factors [24]. Patients were defined as having post-traumatic CP when there was a history of abdominal trauma with imaging evidence of pancreatic injury and subsequent ductal dilation. Hyperlipidemia is considered as an etiology when blood triglyceride is >1000 mg/dL [25] ACP = alcoholic chronic pancreatitis, ICP = idiopathic chronic pancreatitis, CP = chronic pancreatitis.

Exclusion criteria were as follows (Fig 1): pancreatic cancer diagnosed within 2 years after the diagnosis of CP [21], groove pancreatitis (GP) [22], and autoimmune pancreatitis (AIP). In the present study, patients with other etiologies (including abnormal anatomy of pancreatic duct, hereditary, post-traumatic, and hyperlipidemic) were excluded. In the part of study for patients with intraductal pancreatic stones, the patients without pancreatic stone were further excluded.

The study was approved by the Ethics Committee of Changhai Hospital, The Second Military Medical University, Shanghai, China. Written informed consent was obtained from all participating patients. All of the diagnostic and therapeutic modalities were carried out in accordance with the approved guidelines [26,27].

### Definitions

The diagnosis of CP was established when either of the following conditions was met: (1) the presence of pancreatic calcification, (2) pancreatic ductal changes (moderate or marked disease according to Cambridge classification system), (3) abnormal results of pancreatic function tests, (4) endoscopic ultrasound abnormalities indicating CP or (5) histological proof of CP, as described by the Asia-Pacific consensus [28]. Onset of CP was considered when the first manifestation related to CP occurred. Such as recurrent pancreatic pain, chronic pancreatic pain, acute pancreatitis attack, DM, steatorrhea, or asymptomatic patients diagnosed of CP in the course of physical examinations. ACP was considered when alcohol intake exceeded 80 g/day for males or 60 g/day for females for at least 2 years in the absence of other causes [29]. Patients with CP were considered idiopathic when none of the above causes were found.

### Treatment strategy

In our center, endoscopic treatment was taken as principle methods: extracorporeal shockwave lithotripsy (ESWL)/ endoscopic retrograde cholangiopancreatography (ERCP) for stone removal and main pancreatic duct (MPD) drainage [19,30,31]. Surgical treatment (eg, pancreaticoduodenectomy, distal pancreatectomy) was taken when endoscopic treatment is of poor effectiveness or in CP patients with mass. For CP patients who did not experience pain, interventions were performed only when complications such as biliary stricture, infection, or pancreatic pseudocyst (PPC) enlargement had occurred [27], whereas DM and/or steatorrhea were not indications for invasive treatment of CP.

### Statistical analysis

In the comparison of ACP and ICP patients, continuous variables are expressed as mean ± standard deviation (SD) and compared using an unpaired, 2-tailed *t* test or Mann-Whitney test. Categorical variables were compared using the χ^2^ test or Fisher exact test. Cumulative rates of DM, steatorrhea, pancreatic stone, PPC, biliary stricture, and pancreatic cancer after the onset and diagnosis of CP were calculated by using the Kaplan-Meier method [32]. The cumulative rates of DM and steatorrhea after diagnosis of pancreatic stone were also calculated by using the Kaplan-Meier method.

## Results

### General characteristics of study subjects

As shown in Fig 1, from January 2000 to December 2013, a total of 2,287 CP patients were entered in the CP database. After exclusion of 134 patients, which consisted of 16 patients diagnosed with pancreatic cancer within 2 years after the diagnosis of CP, 10 patients diagnosed with GP, and 108 patients diagnosed with AIP, a cohort of 2,153 patients with CP was established. After further exclusion of 64 patients with abnormal anatomy of pancreatic duct, 30 patients with hereditary CP, 10 patients with post-traumatic CP, and 12 patients with hyperlipidemic CP, a cohort of 2,037 patients with CP was finally enrolled.

The general characteristics of these patients are listed in Table 1. The median duration of follow-up was 7.6 (range, 0.0–53.2) years. In the 404 patients of ACP, the median duration of follow-up was 8.3 (range, 0.0–43.2) years, while in the 1,633 patients of ICP, the median duration of follow-up was 7.3 (range, 0.0–53.2) years. Gender, age at the onset and diagnosis of CP, smoking history, pancreatic stones, DM, steatorrhea, PPC, morphology of MPD, and type of pain were significantly different between ACP and ICP patients (all P < 0.001). Initial manifestations, overall treatment, and DM in first-/second-/third-degree relatives were also different between the two groups (all P < 0.05).

**Table 1:** General characteristics of 2,037 patients with CP.

Items	Overall N = 2037	ACP N = 404	ICP N = 1633	P
Male sex	1428 (70.1%)	398 (98.3%)	1031 (63.1%)	<0.001
Age at the onset of CP, y*	38.765±16.468	38.056±17.460	41.631±11.195	<0.001
Age at the diagnosis of CP, y*	43.499±15.436	42.629±16.432	47.015±9.740	<0.001
Adolescent**	250 (12.3%)	2 (0.5%)	248 (15.2%)	<0.001
Smoking history	698 (34.3%)	326 (80.7%)	372 (22.8%)	<0.001
Body mass index*	20.915±3.432	20.970±3.022	20.900±3.539	0.729
Initial manifestations				0.041
Abdominal pain	1700 (83.5%)	354 (87.6%)	1346 (82.4%)	
Endocrine/Exocrine dysfunction	210 (10.3%)	32 (7.9%)	178 (10.9%)	
Others	127 (6.2%)	18 (4.5%)	109 (6.7%)	
Pancreatic stones#	1531 (75.2%)	339 (83.9%)	1192 (73.0%)	<0.001
Age at pancreatic stone diagnosis*	41.813±15.296	47.324±9.458	40.246±16.250	<0.001
Time between onset and pancreatic stone*	5.706±7.196	6.069±6.573	5.602±7.363	0.292
DM	587 (28.8%)	157 (38.9%)	430 (26.3%)	<0.001
Age at diabetes*	46.356±11.470	45.813±9.093	46.554±12.226	0.428
Time between onset and DM*	5.036±7.268	5.685±6.259	4.799±7.596	0.191
Steatorrhea	459 (22.5%)	120 (29.7%)	339 (20.8%)	<0.001
Age at steatorrhea*	42.762±12.653	45.867±8.925	41.774±13.489	<0.001
Time between onset and steatorrhea*	5.005±8.308	5.502±5.839	4.847±8.953	0.370
Biliary stricture	331 (16.2%)	72 (17.8%)	259 (15.9%)	0.339
Age at CBD stenosis*	51.543±12.918	52.118±13.662	49.476±9.587	0.063
Time between onset and CBD stenosis*	5.635±8.712	5.419±8.913	6.412±7.956	0.393
Pancreatic pseudocyst	334 (16.4%)	94 (23.3%)	240 (14.7%)	<0.001
Age at pseudocyst*	46.188±15.070	47.745±9.859	45.570±16.671	0.158
Time between onset and pseudocyst formation*	4.936±6.976	6.666±5.766	4.250±7.299	0.002
Pancreatic Cancer	21 (1.0%)	3 (0.7%)	18 (1.1%)	0.522
Death	70 (3.4%)	13 (3.2%)	57 (3.5%)	0.788
Morphology of MPD				<0.001
Pancreatic stone alone	563 (27.6%)	108 (26.7%)	455 (27.9%)	
MPD stenosis alone	570 (28.0%)	73 (18.1%)	497 (30.4%)	
MPD stenosis and stone	678 (33.3%)	159 (39.4%)	519 (31.8%)	
Complex pathologic changes	226 (11.1%)	64 (15.8%)	162 (9.9%)	
Type of pain				<0.001
Recurrent acute pancreatitis	635 (31.2%)	148 (36.6%)	487 (29.8%)	
Recurrent pain	603 (29.6%)	89 (22.0%)	514 (31.5%)	
Recurrent acute pancreatitis and pain	545 (26.8%)	126 (31.2%)	419 (25.7%)	
Chronic pain	98 (4.8%)	19 (4.7%)	79 (4.8%)	
Without pain	156 (7.7%)	22 (5.4%)	134 (8.2%)	
Severe acute pancreatitis	65 (3.2%)	10 (2.5%)	55 (3.4%)	0.361
Successful drainage+	1403 (68.9%)	284 (70.3%)	1119 (68.5%)	0.491
Overall treatment				0.035
Endotherapy alone	1403 (68.9%)	298 (73.8%)	1105 (67.7%)	
Surgery alone	241 (11.8%)	39 (9.7%)	202 (12.4%)	
Both endotherapy and surgery	172 (8.4%)	36 (8.9%)	136 (8.3%)	
Conservative treatment	221 (10.8%)	31 (7.7%)	190 (11.6%)	
DM in first-/second-/third-degree relatives	116 (5.7%)	35 (8.7%)	81 (5.0%)	0.004
Pancreatic diseases in first-/second-/third-degree relatives (excluding hereditary CP)	31 (1.5%)	8 (2.0%)	23 (1.4%)	0.401

CP = chronic pancreatitis, DM = diabetes mellitus, ICP = idiopathic chronic pancreatitis, ACP = alcoholic chronic pancreatitis, HCP = hereditary chronic pancreatitis

*Mean ± SD.

^#^Pancreatic calcifications were also regarded as stones that are located in branch pancreatic duct or ductulus.

^+^Patients with successful main pancreatic duct (MPD) drainage are those whose CP was established after ERCP or pancreatic surgery or those who underwent successful MPD drainage during administration when CP diagnosis was established.

**Adolescents were patients with CP onset before 18 years old.

### Cumulative rates in ACP and ICP

#### Cumulative rates of DM

**Fig 2:**
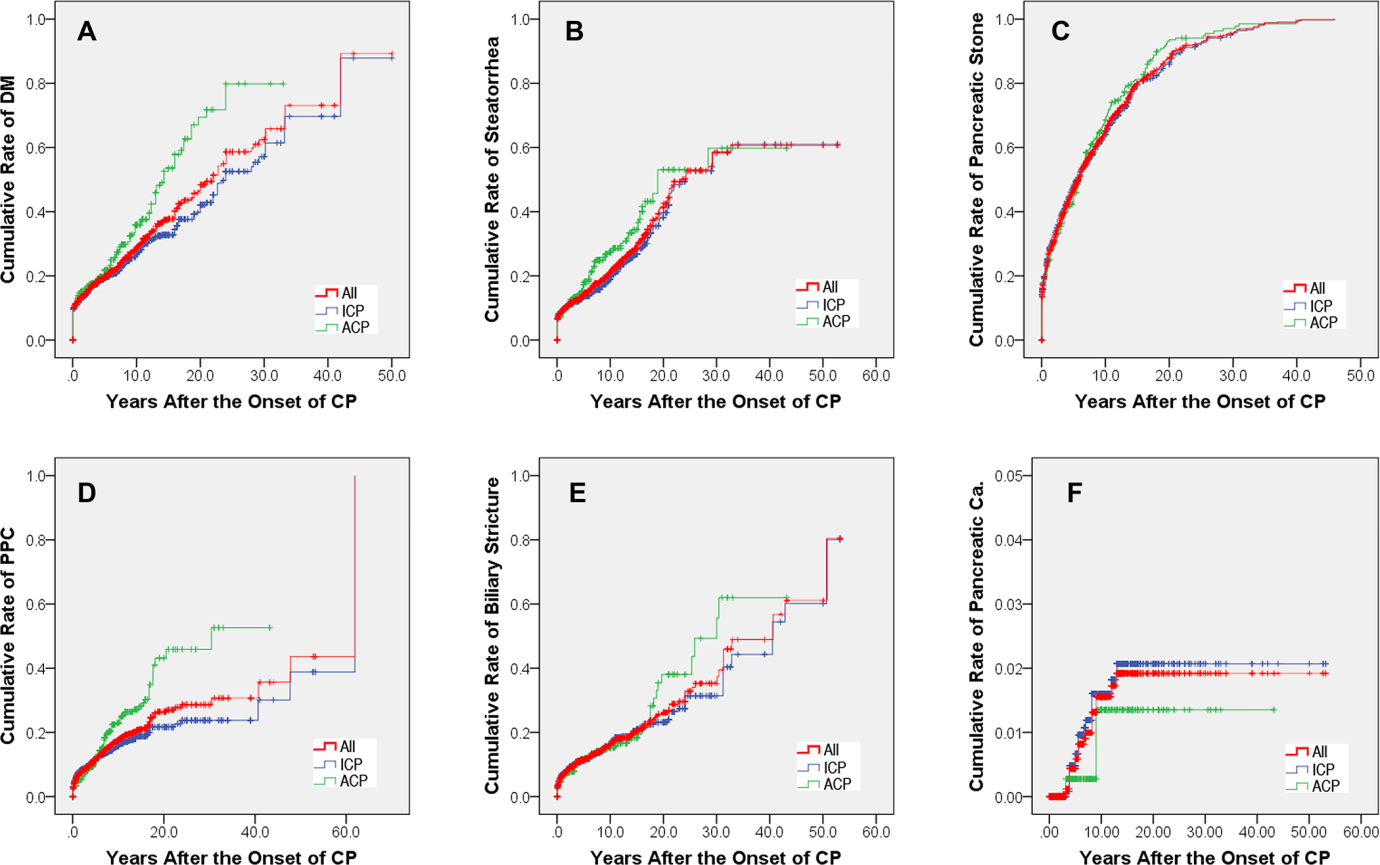
The cumulative rates after the onset of CP. (A) The cumulative rates of diabetes mellitus; (B) The cumulative rates of steatorrhea; (C) The cumulative rates of pancreatic stone; (D) The cumulative rates of pancreatic pseudocysts; (E) The cumulative rates of biliary stricture; (F) The cumulative rates of pancreatic cancer. ACP = alcoholic chronic pancreatitis, Ca. = cancer, CP = chronic pancreatitis, DM = diabetes mellitus, ICP = idiopathic chronic pancreatitis, PPC = pancreatic pseudocyst.

DM developed in 28.8% (587/2,037) eligible patients in the present study. The rates were 38.9% (157/404) in ACP patients and 26.3% (430/1,633) in ICP patients. DM developed in 71, 84 and 120 patients at 3, 5 and 10 years in ACP group after the onset of CP, with the cumulative rates of 17.6% (95% confidence interval [CI]: 15.7%-19.4%), 20.8% (95% CI: 18.7%-22.9%) and 29.7% (95% CI: 27.0%-32.4%); while in 264, 294 and 355 patients in ICP group, with the cumulative rates of 16.2% (95% CI: 15.2%-17.1%), 18.0% (95% CI: 17.0%-19.0%) and 21.7% (95% CI: 20.5%-23.0%), respectively. Significant difference in the rates of DM after the onset of CP was observed between ACP and ICP patients (*P* < 0.001, Fig 2A).

**Fig 3:**
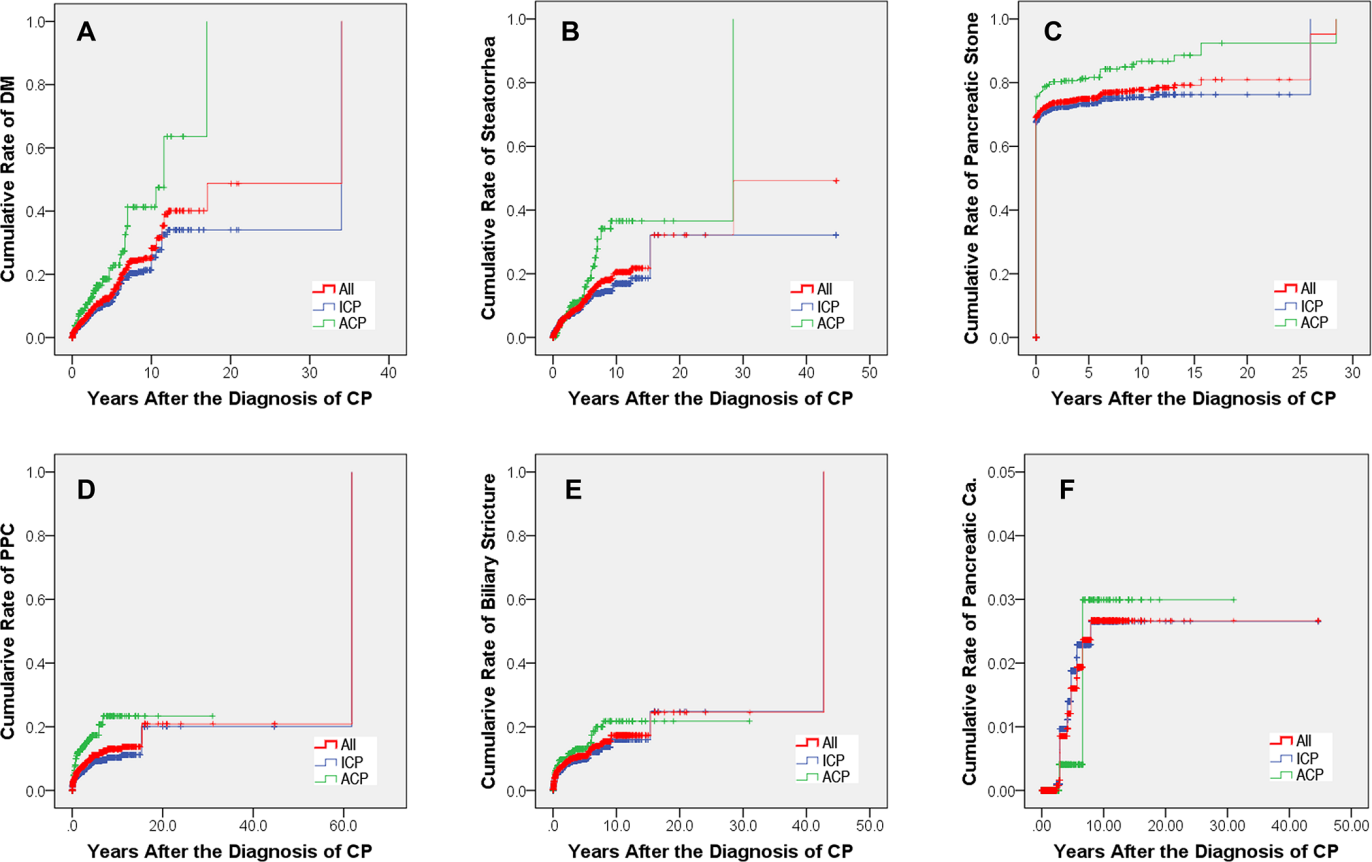
The cumulative rates after the diagnosis of CP. (A) The cumulative rates of diabetes mellitus; (B) The cumulative rates of steatorrhea; (C) The cumulative rates of pancreatic stone; (D) The cumulative rates of pancreatic pseudocysts; (E) The cumulative rates of biliary stricture; (F) The cumulative rates of pancreatic cancer. ACP = alcoholic chronic pancreatitis, Ca. = cancer, CP = chronic pancreatitis, DM = diabetes mellitus, ICP = idiopathic chronic pancreatitis, PPC = pancreatic pseudocyst.

DM developed in 42, 51 and 66 patients at 3, 5 and 10 years in ACP group after the onset of CP, with the cumulative rates of 13.4% (95% CI: 11.3%-15.6%), 15.9% (95% CI: 13.1%-19.0%) and 20.6% (95% CI: 14.6%-26.6%); while in 95, 113 and 152 patients in ICP group, with the cumulative rates of 6.9% (95% CI: 6.1%-7.8%), 8.3% (95% CI: 7.3%-9.2%) and 11.1% (95% CI: 9.4%-12.9%), respectively. Significant difference in the rates of DM after the diagnosis of CP was observed between ACP and ICP patients (*P* < 0.001, Fig 3A).

#### Cumulative rates of steatorrhea

Steatorrhea developed in 22.5% (459/2,037) eligible patients in the present study. The rates were 29.7% (120/404) in ACP patients and 20.8% (339/1,633) in ICP patients. Steatorrhea developed in 51, 64 and 90 patients at 3, 5 and 10 years in ACP group after the onset of CP, with the cumulative rates of 12.9% (95% CI: 11.2%-14.6%), 16.2% (95% CI: 14.2%-18.2%) and 22.8% (95% CI: 20.2%-25.3%); while in 185, 205 and 251 patients in ICP group, with the cumulative rates of 11.4% (95% CI: 10.6%-12.2%), 12.6% (95% CI: 11.7%-13.5%) and 15.4% (95% CI: 14.3%-16.6%), respectively. Significant difference in the rates of steatorrhea after the onset of CP was observed between ACP and ICP patients (*P* = 0.007, Fig 2B).

Steatorrhea developed in 28, 36 and 51 patients at 3, 5 and 10 years in ACP group after the onset of CP, with the cumulative rates of 8.3% (95% CI: 6.6%-10.1%), 10.7% (95% CI: 8.1%-13.4%) and 15.2% (95% CI: 10.0%-20.4%); while in 90, 112 and 130 patients in ICP group, with the cumulative rates of 6.3% (95% CI: 5.5%-7.1%), 7.8% (95% CI: 6.8%-8.9%) and 9.1% (95% CI: 7.3%-10.9%), respectively. Significant difference in the rates of steatorrhea after the diagnosis of CP was observed between ACP and ICP patients (*P* = 0.002, Fig 3B).

#### Cumulative rates of pancreatic stone

Pancreatic stone developed in 75.2% (1,531/2,037) eligible patients in the present study. The rates were 83.9% (339/404) in ACP patients and 73.0% (1,192/1,633) in ICP patients. Pancreatic stone developed in 140, 172 and 266 patients at 3, 5 and 10 years in ACP group after the onset of CP, with the cumulative rates of 34.7% (95% CI: 32.3%-37.0%), 42.6% (95% CI: 40.1%-45.0%) and 65.8% (95% CI: 63.5%-68.2%); while in 606, 744 and 945 patients in ICP group, with the cumulative rates of 37.1% (95% CI: 35.9%-38.3%), 45.6% (95% CI: 44.3%-46.8%) and 57.9% (95% CI: 56.6%-59.1%), respectively. No significant difference in the rates of pancreatic stone after the onset of CP was observed between ACP and ICP patients (*P* = 0.194, Fig 2C).

Pancreatic stone developed in 324, 326 and 336 patients at 3, 5 and 10 years in ACP group after the onset of CP, with the cumulative rates of 95.6% (95% CI: 93.6%-97.5%), 96.2% (95% CI: 94.2%-98.1%) and 99.1% (95% CI: 97.1%-99.6%); while in 1,166, 1,174 and 1,187 patients in ICP group, with the cumulative rates of 97.8% (95% CI: 96.7%-98.9%), 98.5% (95% CI: 97.4%-99.6%) and 99.6% (95% CI: 98.4%-99.6%), respectively. Significant difference in the rates of pancreatic stone after the diagnosis of CP was observed between ACP and ICP patients (*P* < 0.001, Fig 3C).

#### Cumulative rates of PPC

PPC developed in 16.4% (334/2,037) eligible patients in the present study. The rates were 23.3% (94/404) in ACP patients and 14.7% (240/1,633) in ICP patients. PPC developed in 32, 43 and 74 patients at 3, 5 and 10 years in ACP group after the onset of CP, with the cumulative rates of 7.9% (95% CI: 6.6%-9.3%), 10.7% (95% CI: 9.0%-12.3%) and 18.4% (95% CI: 16.0%-20.7%); while in 145, 175 and 211 patients in ICP group, with the cumulative rates of 8.9% (95% CI: 8.2%-9.6%), 10.7% (95% CI: 10.0%-11.5%) and 12.9% (95% CI: 11.9%-14.0%), respectively. Significant difference in the rates of PPC after the onset of CP was observed between ACP and ICP patients (*P* = 0.001, Fig 2D).

PPC developed in 50, 55 and 61 patients at 3, 5 and 10 years in ACP group after the onset of CP, with the cumulative rates of 13.5% (95% CI: 11.6%-15.3%), 14.8% (95% CI: 12.7%-17.0%) and 16.4% (95% CI: 13.3%-19.6%); while in 87, 104 and 107 patients in ICP group, with the cumulative rates of 5.8% (95% CI: 5.1%-6.5%), 6.9% (95% CI: 6.0%-7.8%) and 7.1% (95% CI: 6.1%-8.2%), respectively. Significant difference in the rates of PPC after the diagnosis of CP was observed between ACP and ICP patients (*P* < 0.001, Fig 3D).

#### Cumulative rates of biliary stricture

Biliary stricture developed in 16.2% (331/2,037) eligible patients in the present study. The rates were 17.8% (72/404) in ACP patients and 15.9% (259/1,633) in ICP patients. Biliary stricture developed in 31, 45 and 90 patients at 3, 5 and 10 years in ACP group after the onset of CP, with the cumulative rates of 7.7% (95% CI: 6.4%-8.9%), 11.1% (95% CI: 9.6%-12.7%) and 13.4% (95% CI: 11.4%-15.3%); while in 150, 172 and 214 patients in ICP group, with the cumulative rates of 9.2% (95% CI: 8.5%-9.9%), 10.5% (95% CI: 9.7%-11.1%) and 13.1% (95% CI: 14.2%-12.0%), respectively. No significant difference in the rates of biliary stricture after the onset of CP was observed between ACP and ICP patients (*P* = 0.609, Fig 2E).

Biliary stricture developed in 42, 44 and 51 patients at 3, 5 and 10 years in ACP group after the onset of CP, with the cumulative rates of 11.1% (95% CI: 6.6%-10.1%), 11.5% (95% CI: 9.6%-13.4%) and 13.3% (95% CI: 9.9%-16.8%); while in 117, 122 and 141 patients in ICP group, with the cumulative rates of 7.7% (95% CI: 6.9%-8.5%), 8.1% (95% CI: 7.2%-8.9%) and 9.3% (95% CI: 7.6%-11.0%), respectively. Significant difference in the rates of biliary stricture after the diagnosis of CP was observed between ACP and ICP patients (*P* = 0.040, Fig 3E).

#### Cumulative rates of pancreatic cancer

Pancreatic cancer developed in 1.0% (21/2,037) eligible patients in the present study. The rates were 0.7% (3/404) in ACP patients and 1.1% (18/1,633) in ICP patients. Pancreatic cancer developed in 0, 1 and 3 patients at 3, 5 and 10 years in ACP group after the onset of CP, with the cumulative rates of 0.0%, 0.2% (95% CI: 0.0%-0.5%) and 0.7% (95% CI: 0.0%-1.5%); while in 0, 6 and 16 patients in ICP group, with the cumulative rates of 0.0%, 0.4% (95% CI: 0.2%-0.6%) and 1.0% (95% CI: 0.6%-1.4%), respectively. No significant difference in the rates of pancreatic cancer after the onset of CP was observed between ACP and ICP patients (*P* = 0.404, Fig 2F).

Pancreatic cancer developed in 1, 1 and 3 patients at 3, 5 and 10 years in ACP group after the onset of CP, with the cumulative rates of 0.2% (95% CI: 0.0%-0.6%), 0.2% (95% CI: 0.0%-0.6%) and 0.7% (95% CI: 0.0%-2.5%); while in 9, 15 and 18 patients in ICP group, with the cumulative rates of 0.6% (95% CI: 0.3%-0.8%), 0.9% (95% CI: 0.4%-1.4%) and 1.1% (95% CI: 0.4%-1.8%), respectively. No significant difference in the rates of pancreatic cancer after the diagnosis of CP was observed between ACP and ICP patients (*P* = 0.569, Fig 3F).

### Endocrine and exocrine impairment in patients with pancreatic stones

After further exclusion of 506 patients without pancreatic stones, a cohort of 1,531 patients was included in the present study, which contains 339 of ACP patients and 1,192 of ICP patients.

#### Endocrine impairment in patients with pancreatic stones

**Fig 4:**
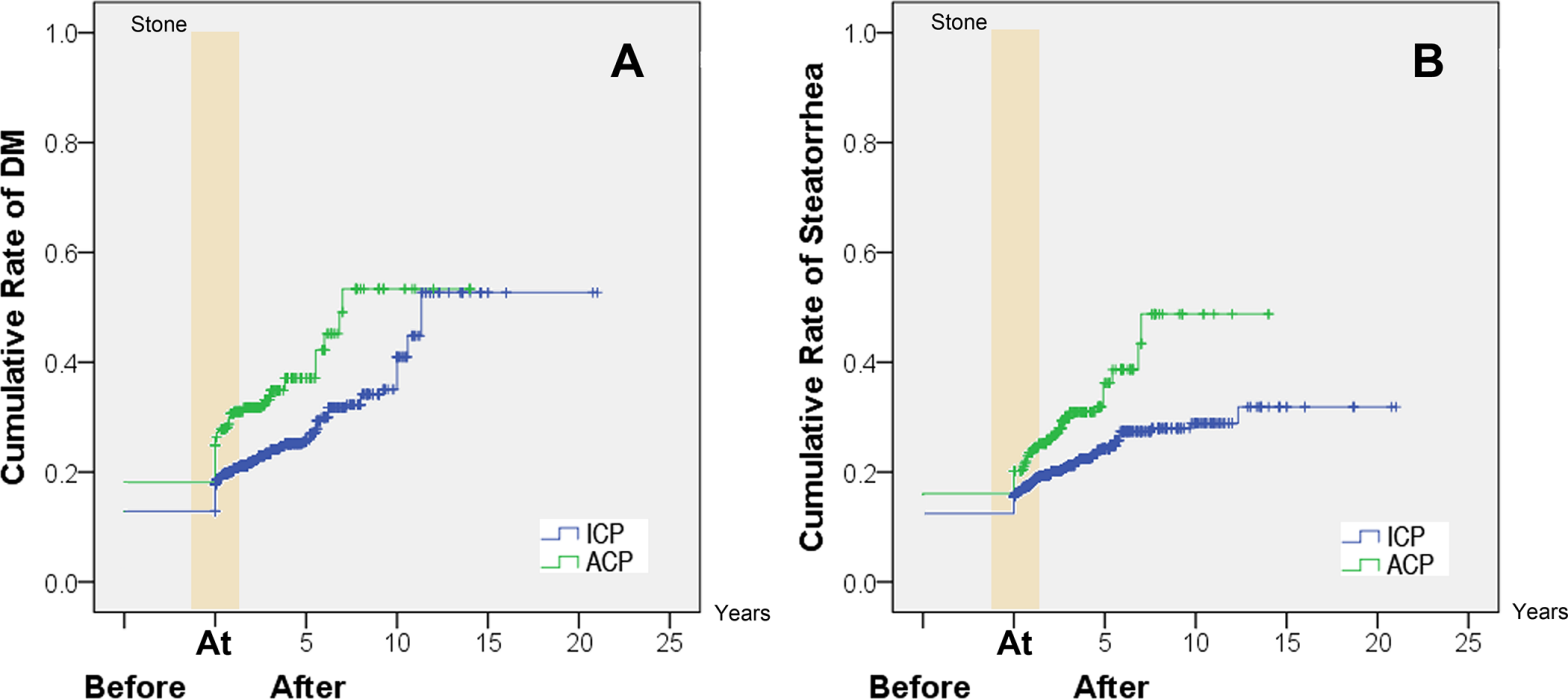
The cumulative rates after the diagnosis of pancreatic stone. (A) The cumulative rates of diabetes mellitus; (B) The cumulative rates of steatorrhea. ACP = alcoholic chronic pancreatitis, DM = diabetes mellitus, ICP = idiopathic chronic pancreatitis.

DM developed in 37.2% (126/339) patients in ACP group and 26.2% (312/1,192) in ICP patients. DM developed in 62, 116 and 126 patients before pancreatic stone diagnosis, 5 and 10 years after pancreatic stone diagnosis in ACP group, with the cumulative rates of 18.3% (95%CI: 16.2%-20.3%), 34.2% (95% CI: 31.4%-37.1%) and 37.2% (95% CI: 32.1%-42.3%); while in 155, 273 and 298 patients in ICP group, with the cumulative rates of 13.0% (95%CI: 12.0%-14.0%), 22.9% (95% CI: 21.5%-24.3%) and 25.0% (95% CI: 22.6%-27.4%), respectively. Significant difference in the rates of DM development in patients with pancreatic stones was observed between ACP and ICP patients (*P* < 0.001, Fig 4A).

#### Exocrine impairment in patients with pancreatic stones

Steatorrhea developed in 31.6% (107/339) patients in ACP group and 21.6% (258/1,192) in ICP patients. Steatorrhea developed in 54, 101 and 107 patients before pancreatic stone diagnosis, 5 and 10 years after pancreatic stone diagnosis in ACP group, with the cumulative rates of 15.9% (95%CI: 14.0%-17.9%), 27.8% (95% CI: 26.5%-33.1%) and 31.6% (95% CI: 26.0%-37.1%); while in 142, 245 and 257 patients in ICP group, with the cumulative rates of 11.9% (95%CI: 11.0%-12.8%), 20.6% (95% CI: 19.2%-21.9%) and 21.6% (95% CI: 19.5%-22.6%), respectively. Significant difference in the rates of steatorrhea development in patients with pancreatic stones was observed between ACP and ICP patients (*P* < 0.001, Fig 4B).

## Discussion

To our knowledge, this is a study about natural course of ACP and ICP based on the largest cohort of CP patients. In the present study, epidemiological features, initial manifestations, natural course and complications of CP were evaluated.

The etiologies of CP were significantly different in different countries or time period (Table 2). ACP was considered the most frequent etiology of CP in western countries, which account for about 49.1%-89.7% [13,33–35]. ACP was also the most frequent etiology of CP in Japan, accounting for 67.5% [36]. However, the etiology of CP in developing countries of Asia is not the same. ICP was reported only 12.9% in 1994–2004 in China [11], but was reported the most frequent etiology of CP in recent studies in China and India, accounting for up to 57.3%-69.6% [11,20,37–41]. In the present study, ICP was defined in 75.8% patients.

**Table 2:** Researches on etiologies of CP patients in different countries.

Countries	Inclusion period	ACP	ICP	Other etiologies
Danish [33]	1995–2010	6306 (52.7%)	-	5666 (47.3%)*
Japan [36]	2011	1171 (67.5%)	347 (20.0%)	216 (12.5)
China [11]	1994–2004	705 (35.1%)	259 (12.9%)	1044 (52.0%)
The United States [34]	2000–2014	575 (49.1%)	286 (24.4%)	310 (26.5%)
India [39]	-2007	400 (38.7%)	622 (60.2%)	11 (1.1%)
Brazil [35]	1963–1986	282 (85.7%)	34 (10.3%)	13 (4.0%)
Germany [13]	1989–1995	733 (72.0%)	224 (22.0%)	61 (6.0%)
Present study	2000–2013	404 (18.8%)	1633 (75.8%)	116 (5.4)

*Nonalcoholic CP

ACP = alcoholic chronic pancreatitis, CP = chronic pancreatitis, ICP = idiopathic chronic pancreatitis

The data of this study reveal a significantly difference between ACP and ICP patients. ACP is likely to occur in male patients with smoking history. Age at onset and diagnosis of ACP is significantly younger than ICP patients. It was reported that the younger the patients are when they begin their alcohol abuse, the shorter the time required for chronic pancreatitis to develop seems to be [42]; while ICP has two age peaks, one in young patients and the other in elderly patients [43]. ACP patients are more likely to have recurrent acute pancreatitis attack, while ICP patients have more recurrent pain or without pain [9,44,45]. It is generally assumed that alcohol dependent develop clinically acute pancreatitis in the context of preexistent (clinically latent) CP with distinct fibrosis and protein plugs in the small ducts [46], which seriously affect the life quality of these patients.

The development of DM, steatorrhea, and PPC after the onset and diagnosis of CP were significantly different between ACP and ICP patients. The development of pancreatic stone and biliary stricture were also different after the diagnosis of CP. The insufficiencies of pancreatic endocrine and exocrine were more common, as well as occurred earlier, in ACP patients, which reveals the progressive pancreatic dysfunction. The development of PPC was also more common and earlier in ACP patients, which may related to the recurrent episodes of acute pancreatitis attack [8]. Although there’s no significant difference in the development of pancreatic stone and biliary stricture after the onset of CP, pancreatic stone and biliary stricture developed much faster in ACP patients after the diagnosis of CP [43]. As for pancreatic cancer development, no significant difference was observed between ACP and ICP patients. In consideration of endocrine and exocrine destruction, PPC, biliary stricture, and pancreatic stone development, it seems ACP patients usually have a more severe course of CP.

The development of DM and steatorrhea were compared between ACP and ICP after the diagnosis of pancreatic stone. The incidence of steatorrhea in ACP patients increased steeply from 15.9% before to 27.8% within 5 years from onset of pancreatic stone. This indicates a rather close correlation between exocrine insufficiency and pancreatic stone. However, the correlation was much less marked in ICP patients since onset of pancreatic stone preceded exocrine insufficiency in 9.7% of patients by up to 10 years. A similar phenomenon can be observed about pancreatic endocrine insufficiency. Thus it appears that the dynamics of progression of endocrine and exocrine insufficiency and of pancreatic stones differ markedly between ACP and ICP patients.

Our study has some limitations. First, the retrospectively acquired data collected between 2000 and 2004 might introduce recall bias. Nevertheless, statistical analysis showed that there was no significant difference between the clinical characteristics of patients admitted before and after January 2005. In this sense, the recall bias minimally influenced the results of the study. Second, 603 CP patients were followed up for less than 2 years after the diagnosis of CP; among these patients, several pancreatic cancer patients may have been misdiagnosed as CP. However, these limitations minimally influence the results considering the relatively large sample size of the study.

In conclusion, ACP and ICP have many features in common. However, the long-term profile of these two groups differs in some important aspects, particularly in the pain attack, morphology, and development of complications. It seems ACP patients usually have a more severe course of CP. These differences should be recognized in the diagnosis and treatment of CP. Lifestyle modification like abstinence may benefit the ACP patients.

## Abbreviations

ACP: alcoholic chronic pancreatitis
AIP: autoimmune pancreatitis
CI: confidence interval
CP: chronic pancreatitis
CT: computed tomography
DM: diabetes mellitus
ERCP: endoscopic retrograde cholangiopancreatography
ESWL: extracorporeal shockwave lithotripsy
GP: groove pancreatitis
ICP: idiopathic chronic pancreatitis
MPD: main pancreatic duct
MRI: magnetic resonance imaging
PPC: pancreatic pseudocyst
SD: standard deviation
